# Functional and structural abnormalities of the kidney and urinary tract in severely malnourished children - A hospital based study

**DOI:** 10.12669/pjms.325.10457

**Published:** 2016

**Authors:** Misbah Anjum, Khemchand N Moorani, Ifra Sameen, Muhammad Ayaz Mustufa, Shazia Kulsoom

**Affiliations:** 1 Misbah Anjum, MBBS, FCPS. Assistant Professor, Department of Pediatric Medicine (Unit-III), National Institute of Child Health (NICH), Jinnah Sindh Medical University (JSMU), Karachi, Pakistan; 2 Khemchand N Moorani, FCPS, MCPS, MBBS. Professor, Pediatric Nephrology & Medical Unit-III, National Institute of Child Health (NICH), Jinnah Sindh Medical University (JSMU), Karachi, Pakistan; 3 Ifra Sameen, MBBS, MCPS, FCPS. Associate Professor, Department of Pediatric Medicine (Unit-III), National Institute of Child Health (NICH), Jinnah Sindh Medical University (JSMU), Karachi, Pakistan; 4 Muhammad Ayaz Mustufa, MBE, PhD. Pakistan Health Research Centre (PHRC), Specialized Research Centre on Child Health, Senior Research Officer/Centre In-charge, National Institute of Child Health (NICH), Jinnah Sindh Medical University (JSMU), Karachi, Pakistan; 5 Shazia Kulsoom, MBBS FCPS. Assistant Professor, Department of Pediatric Medicine (Unit-III), National Institute of Child Health (NICH), Jinnah Sindh Medical University (JSMU), Karachi, Pakistan

**Keywords:** Glomerular filtration rate, Severe acute malnutrition, Urinary tract abnormalities

## Abstract

**Objectives:**

The association of malnutrition and systemic diseases like chronic kidney disease (CKD) is well known. Various urinary tract abnormalities may be associated with malnutrition. So objective of current study was to determine the frequency of functional and structural urinary tract abnormalities in severely malnourished children admitted in Nutritional Rehabilitation Unit (NRU) of a tertiary care facility, Karachi.

**Methods:**

This descriptive cases series of 78 children was conducted in NRU from October 2014 - March 2015. All newly admitted children aged 2-60 months, diagnosed as Severe Acute Malnutrition (SAM) were studied and children with known kidney and urinary tract disorders were excluded. Detailed history, examination and investigations like serum creatinine, ultrasound kidney and urinary tract in addition to routine tests for SAM, were done. A proforma was used to collect demographic data, clinical history, physical findings, and radio-imaging and biochemical investigations. Glomerular filtration rate (GFR) was calculated using Schwartz equation. Data was analyzed using descriptive statistics.

**Results:**

Among 78 children, male to female ratio was equal. Mean age was 18±15.53 months and majority (79.48%) of children were below 24 months. Majority (82%) of children with SAM had marasmus whereas 18% had edematous malnutrition. Out of 78, 57 (73%) children had either functional (80.7%) and or structural (19.3%) abnormalities whereas 21(36.84%) had normal functional and structural status. Most common functional abnormality was subnormal GFR (<90ml/min/1.73 m^2^) found in all 46 children. Functional abnormities were more common in children below 24 months. Other functional disorders were Bartter syndrome, renal tubular acidosis and urinary tract infection (UTI) found in two cases each. Common structural abnormalities were echogenic kidneys (n=4, 36%), hydronephrosis (n=3, 27%), hypoplastic kidneys (n=3, 27%) and calculi (n=1, 9%). Subnormal GFR was also found in all cases with structural abnormalities. UTI was observed exclusively in two children among 11 with structural abnormalities.

**Conclusion:**

A high frequency of functional abnormalities and noticeable proportion of structural abnormalities of urinary tract were detected in children with SAM. Current finding suggest that multicenter study at national level may be undertaken to generate better data about prevalence of renal diseases in SAM.

## INTRODUCTION

Functional kidney disorders and structural urinary tract abnormalities are the significant problems in pediatric age group. These may result in recurrent infections, growth failure, edema, proteinuria and hematuria requiring hospitalization for investigation and medical or surgical treatment. Certain congenital abnormalities like posterior urethral valve, primary vesicoureteral reflux and hypo-dysplastic /multi-cystic disorders may end up in chronic kidney disease (CKD)/end stage renal disease requiring renal replacement therapy in the form of either dialysis or transplantation.^1^-^4^

Sever acute malnutrition (SAM) is a common contributing factor for childhood morbidity and mortality. SAM accounted for more than 53% of under five mortalities due to pneumonia, diarrhea, malaria and perinatal illnesses.^5^ Under-nutrition is a major problem in Pakistan and a very high rates of underweight (31.5%), stunting (43.7%) and wasting (16.8%) are prevalent in children below 5 years of age.^5^,^6^

There is an association of malnutrition and various kidney diseases which may be either due to primary deficiency of nutrients or secondary to kidney problem. In developing countries, it is mostly primary malnutrition, however a wide range of prevalence of secondary malnutrition (6%-51%) has been reported in hospitalized children.^4^,^5^ Systemic diseases for childhood malnutrition like cystic fibrosis, congenital heart diseases and CKD are well known. Children with kidney diseases are at high risk of malnutrition because of multiple reasons including insufficient food intake, metabolic acidosis, hypermetabolism due to persistent inflammation, hormonal disturbance, increased inflammatory cytokines as in recurrent urinary tract infections.^7^-^9^ Anemia and secondary hyperparathyroidism also contribute for malnutrition significantly in CKD.^8^,^9^

Diagnosis of kidney disease and urinary tract abnormalities in malnourished children is important to identify reversible causes which may progress to advanced chronic kidney disease.^1^,^4^ At times it becomes difficult to decide whether malnutrition is primary and kidney problems are secondary or vice versa. Though, history and physical findings are more specific in kidney disorders like in obstructive uropathies but at times functional disorders with hypoplastic kidneys, tubular disorders and low grade vesicoureteral reflux may remain silent and present with growth failure and malnutrition.^4^

Studies have shown that malnutrition is common associated feature with various renal disorders particularly CKD, obstructive uropathies and tubular disorders (like renal tubular acidosis and Bartter syndrome) but the literature is scanty regarding prevalence of kidney and urinary tract abnormalities in malnourished children.^1^-^4^

So we carried out this study with the objectives to determine frequency of functional and structural abnormalities of kidney and urinary tract in children with SAM admitted in NRU of National Institute of Child Health (NICH), Karachi.

## METHODS

This descriptive cases series was carried out in the NRU of Pediatric Medical Ward–III, NICH, Karachi from October 2014 - March 2015. Seventy-eight children with SAM (either marasmus or kwashiorkor) were studied. Ethical approval was taken from Institutional Ethical Review Committee. Informed written consent was obtained from parents or care givers before enrollment in the study. All patients with SAM, who were admitted in NRU during study period and fulfilled the inclusion criteria, were studied.

### Inclusion Criteria

All children aged 2-60 months, of either gender, who were admitted first time in NRU and fulfilled the diagnosis of SAM as defined by presence of either bilateral pitting edema or weight for height z-score <-3, were enrolled.^10^

### Exclusion Criteria

All children with already known kidney problem or symptomatic congenital anomalies of kidney and urinary tract (CAKUT) and functional (CKD, glomerular or tubular diseases, urinary tract infection) abnormalities were excluded. Children with SAM who were either readmitted for various problems or left before completion of lab workup were excluded.

All children were managed according to National Integrated Management of Neonatal and Childhood Illnesses (IMNCI) guidelines for SAM and associated infections and other complications like hypothermia and hypoglycemia.^11^ Immunization status was considered as complete if vaccinated according to WHO schedule.^12^

After detailed history and physical examination, baseline laboratory investigations including complete blood count (CBC), creatinine (Cr), serum electrolytes, urinalysis and culture; and Ultrasonography (US) of kidneys, ureter and bladder (KUB) was done for kidney size, echogenicity, hydronephrosis, hydroureter and cyst or stone in the kidneys and urinary tract. Functional status of kidneys was assessed by estimating glomerular filtration rate (eGFR) and urinalysis for evidence of proteinuria, hematuria, pyuria and culture. Serum Cr was done by Jaffey’s method and GFR was estimated by Schwartz formula.^13^ Based on eGFR, functional renal status was classified as normal or subnormal and eGFR less than 90 ml/min per 1.73 m^2^ was considered as subnormal.^13^ Subnormal eGFR was further categorized in to 4 groups based on severity of reduction in eGFR.^14^ Children with renal abnormalities were divided into two major groups. Functional abnormality was defined as subnormal GFR (< 90 ml/min /1.73 m^2^) or tubular, glomerular abnormality (like proteinuria) and or urinary tract infection. Structural abnormalities were defined as either abnormal size of kidneys for age, raised echogenicity, hydronephrosis/hydroureter or urinary tract obstruction due to either congenital or acquired cause for example stone disease.

### Data collection and analysis

Data including sociodemographic, observed complications like pneumonia, diarrhea and physical findings like anthropometry, edema, MUAC (mid upper arm circumference) and laboratory findings was collected on predesigned proforma. Data was computed and analyzed on SPSS version 16 using descriptive statistics. Categorical variables were represented by frequency and percentages whereas quantitative variables were represented by mean ±SD.

## RESULTS

A total of 78 children with SAM were evaluated for various kidney and urinary tract abnormalities. Majority (82%) of children with SAM were marasmus whereas 18% children had edematous malnutrition. Among 78 children studied, 57(73%) had either functional (n=46, 80.7%) or structural abnormalities (n=11, 19.3%) whereas 21 (26.92%) children had normal structural and functional status.

Table-I shows sociodemographic and anthropo-measurements in children with SAM. Mean age was 18±15.53 months and majority (79.48%) of children were below 2 years. Mean weight and height was 5.69±2.42 kg and 68.52±13.52 cm respectively.

**Table-I:** Socio-demography and anthropometrics of children with severe acute malnutrition n=78.

*Parameters*	*Structural abnormalities* *N=11 Mean ± SD*	*Functional abnormalities* *N=46 Mean± SD*	*Without abnormalities* *N=21 Mean ± SD*
Age (months)	28.36±20.02	14.65±13.85	19.32±14.50
<24	6 (13±6.69)	41(10.73±7.47)	15(12.40±6.4)
>24	5(46.8±13.68)	5(46.8±12.29)	6(40±6.2)
*Gender*			
Male	7(63.6%)	27(58.7%)	5(22.7%)
Female	4(36.4%)	19(41.3%)	16(72.7%)
Weight(kg)	5.8±2.07	5.07±2.16	6.795±2.8
<24 months	4.68±1.60	4.66±1.85	5.56±2.33
> 24 months	7.14±1.84	8.46±1.47	9.88±0.54
Height(cm)	74.09±12.153	64.87±11.56	73.74±15.96
>24 months	65.5±8.07	62.32±9.11	66.63±13.07
> 24 months	84.4±6.66	85.8±7.66	91.05±5.09
FOC* (cm)	42.5±3.48	39.96±4.24	41.28±3.85
MUAC*(cm)	9.7±0.98	10.048±1.73	10.08±1.48
Bottle feeding	8(72.7%)	21(45.7%)	10(45.5%)
Delayed weaning	6(54.5%)	16(34.8%)	9(40.9%)
Unvaccinated	7(64%)	22(46%)	9(43%)
> 2 siblings < 5 yrs	3(27.3%)	16(34.8%)	6(27.3%)

* FOC=Fronto-occipital circumference, MUAC=mid upper arm circumference.

Among 78 children, 39(50%) were bottle feeder, 38 (48.71%) unvaccinated and delayed weaning was noted in 31 (39.7%). Ten cases (12.8%) were either preterm or low birth weight.

**Fig.1:**
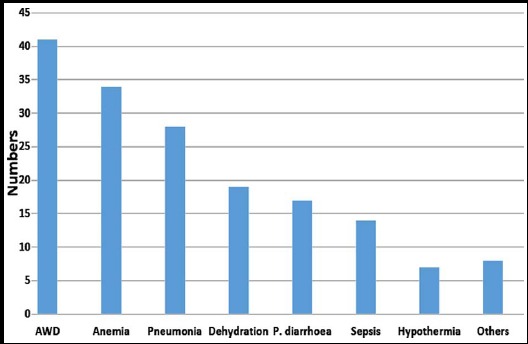
Frequency of complications observed in 78 severely malnourished children. AWD = acute watery diarrhea, P= persistent.

Fig.1 shows complications in children with SAM. Acute watery diarrhea, anemia and pneumonia, were the most common complications accounting for 41(52.6%), 34(43.6%) and 28(35.9%) respectively.

**Fig.2:**
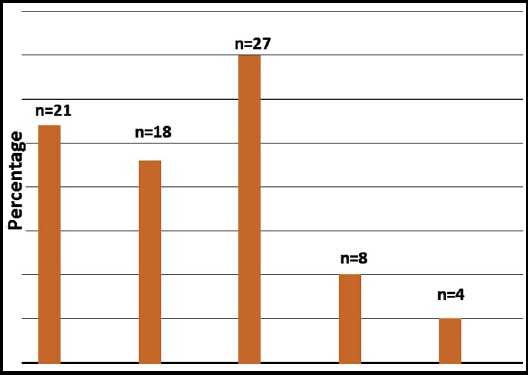
Estimated glomerular filtration rate in 78 children with Severe Acute Malnutrition.

Overall mean eGFR was 71.45±5.65 ml/min/1.73m^2^ and it was normal (> 90 ml/min/1.73m^2^) in 21(27%) children. Severity of functional renal impairment based on eGFR (Fig.2) shows that 27(34.61%) children had eGFR between 30-59 ml, whereas 12(15.38%) had severe functional impairment (GFR<30 ml/min/1.73 m^2^).

Major functional disturbance was subnormal GFR and found in almost all 46(100%) children but more common in children below 24 months (89% compared to children above 24 months (11%). Other functional abnormalities were Bartter syndrome, renal tubular disorder and urinary tract infection in 2 cases (4%) each.

Among 11 children with structural abnormalities, major findings were echogenic kidneys (36%), hydronephrosis (27%), hypoplastic kidneys (27%) and renal calculi (9%). All 11 children with structural renal abnormalities had subnormal GFR and 2(18%) children were complicated with UTI.

Overall laboratory parameters in 78 children with SAM are shown in Table-II. This table shows that mean urea and creatinine levels were 29.0± 17.37 and 0.68 ± 0.48 mg /dl respectively. Mean hemoglobin was 8.66±2.4 G/dl and it was subnormal (<11 G/dl) in 68(87%) patients. Severe anemia (Hb≤6 G/dl) was observed in 9(13%) cases. Table-II also shows that 8 (10%) children had hyponatremia and 9 (11.5%) had hypokalemia. Abnormally low serum bicarbonate (metabolic acidosis) was observed in 10(12.8%) and a high bicarbonate level (metabolic alkalosis) in 2(2.5%) cases.

**Table-II:** Laboratory parameters in 78 children with severe acute malnutrition.

*Parameters*	*Structural abnormalities Mean ±SD* *N=11*	*Functional abnormalities Mean ±SD* *N=46*	*Without Renal Abnormalities* *Mean ±SD* *N=21*
Hb (G/dl)	8.982+11.2	8.51±2.64	8.79±2.366
Anemia (<11)	10(90%)	39(88%)	18(85.7%)
Severe anemia (<6)	–––––	6(13%)	3(14.3%)
Urea(mg/dl)	30.57+18.32	31.37±19.05	23.56±11.215
Cr (mg/dl)	0.973+0.535	0.759±0.49	0.352±0.103
eGFR (ml/min/1.73m^2^	43.76+16	49.68±21.55	133±57
Subnormal (<90)	11(100%)	46(100%)	–––––––
Na(meq/L)	134.73+7.058	138.76±6.085	134.3±6.947
Hyponatremia (<130)	3(27%)	1(2.1%)	4(19%)
K meq/L)	3.23+1.089	3.987±1.08	4.32±1.24
Severe hypokalemia (<2.5)	4(36%)	4(8.7%)	1(4.7%)
Cl (meq/L)	102.5+4.353	104.96±5.25	101.25±7.65
Ca(mg/dl)	8.82+0.73	8.85±1.2	8.88±1
Hypocalcaemia (<7)	–––––––	2(4.3%)	1(4.7%)
HCO_3_(meq/L)	20.15+5.02	20.7±7.34	22.12±2.25
Metabolic acidosis (<20)	3(27%)	20.7±7.34	22.12±2.25
Metabolic alkalosis (>30)	–––––––	2(4.3%)	–––––––
Hypoglycemia (< 55mg/dl)	–––––––	2(4.3%)	3(14.3%)

## DISCUSSION

A high frequency of functional abnormalities and significant proportion of structural abnormalities were detected in our study population. Kidney diseases including urinary tract infections, congenital anomalies of kidneys and urinary tract (CAKUT) and abnormal renal functional status (decreased eGFR) are under reported in malnourished children. To our knowledge this is the first study which has highlighted the magnitude of functional and structural abnormalities in children with SAM.

Severe Acute malnutrition accounts for 53% of under-five mortality.^5^ More than 75% non- communicable diseases (NCDs) deaths occur in developing countries and chronic kidney disease (CKD) is an important contributor of these deaths.^15^,^16^

In the present study, we found that 73% of children had either functional or structural abnormalities. It is surprising that functional renal impairment was found in more than 80% of children studied. This could be explained by multiple factors. Most important one is the fact that though nephron formation is completed by 35-36 weeks but glomerular and tubular growth continues till the age of 18 months and GFR continues to increase to adult level by the age of two years.^17^ Other factors which can explain subnormal GFR in the current study are enrolled participants with history of low birth weight and prematurity (12%), presence of intercurrent infections (20-52%), dehydration (52%) and methods used for assessing serum creatinine and estimating GFR.

Renal growth and GFR are dependent on optimal nutrition and in our study SAM may be the primary denominator which has contributed for impaired plasma flow and decreased GFR.^8^-^10^ In addition, the method used for estimating serum creatinine and GFR in our children may not be reliable in young infants of less than two years of age since majority (79.48%) of children were below years.^13^

We found structural abnormalities of urinary tract in 14% and almost all had CAKUT except one who had stone disease. These cases would otherwise have been missed if not investigated for renal abnormalities and would have ended in CKD. This is evident that in the current study, all children with CAKUT had subnormal GFR. Though we did not follow these patients for three months to label as CKD but most children born with major structural abnormalities like posterior urethral valve with low GFR are generally considered to have CKD without waiting 03 months for labeling such diagnosis.^14^ Harambat J et al. reported CAKUT as the major cause of CKD (48%) followed by hereditary nephropathy (10%) below 21 years of age in United States.^1^,^17^ Though in a study by Gopal G et al, children with CAKUT were excluded but still they found CAKUT in 6.25% of malnourished children.^18^

CAKUT has also been documented in otherwise asymptomatic children on routine screening.^19^ It has also found that CAKUT is being the commonest cause of CKD (58-59%) and end stage renal disease (30-40%) in children.^20^-^23^ Common associated complications in SAM (Fig.1) observed in our study were acute watery diarrhea with dehydration (52%), persistent diarrhea (22%), pneumonia (36%) and sepsis (18%). As pointed earlier on while explaining the reasons for subnormal eGFR, these conditions may have contributed significantly to subnormal GFR, high frequency of anemia (87%), electrolyte disturbance and metabolic abnormalities (Table-II). Evidence of either leucocytosis (19.23%) or leucopenia (12.82%) and thrombocytopenia (24.35%) favours the infectious complications and UTI. Electrolyte abnormalities (10%) like hyponatremia and hypokalemia (10%) in this study could be attributed as primary findings of SAM or diarrheal losses or primary renal tubular disorders. Findings of metabolic acidosis and alkalosis (each in 2 cases) has lead to diagnosis of renal tubular acidosis and Bartter syndrome though children were admitted with primary malnutrition.^24^ Electrolyte abnormalities and Bartter syndrome were also found in our previous study on morbidity patterns in children with SAM. Urinary tract infection (UTI) was found in 5% of children which is similar (3.8%) to our previous study..^24^ A higher percentage (11.34%) of UTI in malnourished children was found by Gopal et al. from our neighboring country in which study they also excluded the CAKUT.^18^ Renal stone disease in current study (1.3%) is similar to our previous one in which we found stone disease in 2.3% of malnourished children.^24^

Findings of significant infectious complications, hematological and electrolyte abnormalities suggest that investigations for functional and structural abnormalities of urinary tract may have been undertaken after stabilization from acute complications.

### Limitations of the study

Limitations are small sample size, tertiary care setting where children were admitted with complications, lack of follow up status after stabilization and treatment of dehydration and infections. Since this study is a single center experience, the results obtained may not be generalized. However, we do strongly believe that our results are important with respect to detection of functional and structural abnormalities in severely malnourished children. It is also difficult to establish that, whether this subnormal GFR is indicator of CKD or transiently decreased renal functions due to one or more than one reversible factors like dehydration and sepsis since we do not have follow up status.

## CONCLUSION

We found a high frequency of functional and structural renal abnormalities in severely malnourished children. However, we recommend that a multicenter study in hospitalized children with SAM may be conducted to generate better data on magnitude of functional and structural abnormalities.
